# Autonomic neuropathy in Fabry disease: a prospective study using the Autonomic Symptom Profile and cardiovascular autonomic function tests

**DOI:** 10.1186/1471-2377-10-38

**Published:** 2010-06-07

**Authors:** Marieke Biegstraaten, Ivo N van Schaik, Wouter Wieling, Frits A Wijburg, Carla EM Hollak

**Affiliations:** 1 Department of Neurology, Academic Medical Center, Amsterdam, the Netherlands; 2 Department of Internal Medicine, Academic Medical Center, Amsterdam, the Netherlands; 3 Department of Pediatrics, Emma Children's Hospital, Academic Medical Center, Amsterdam, the Netherlands; 4 Department of Internal Medicine, Division of Endocrinology and Metabolism, Academic Medical Center, Amsterdam, the Netherlands

## Abstract

**Background:**

Fabry patients have symptoms and signs compatible with autonomic dysfunction. These symptoms and signs are considered to be due to impairment of the peripheral nervous system, but findings indicative of autonomic neuropathy in other diseases, such as orthostatic intolerance and male sexual dysfunction, are infrequently reported in Fabry disease. The aim of our study was to investigate autonomic symptoms and cardiovascular autonomic function in a large cohort of male and female Fabry patients.

**Methods:**

Forty-eight Fabry patients (15 male, 30 treated with enzyme replacement therapy) and 48 sex- and age-matched controls completed a questionnaire on autonomic symptoms (the Autonomic Symptom Profile). Thirty-six Fabry patients underwent cardiovascular function tests.

**Results:**

The Autonomic Symptom Profile revealed a significantly higher sum score in Fabry patients than in healthy control subjects (22 versus 12), but a relatively low score compared to patients with proven autonomic neuropathy. Fabry patients scored worse than healthy controls in the orthostatic intolerance domain. Scores in the male sexual dysfunction domain were comparable between healthy controls and male Fabry patients. The cardiovascular autonomic function tests revealed only mild abnormalities in seven patients. None of these seven patients showed more than one abnormal test result. Enzyme replacement therapy was not associated with less severe disease, lower ASP scores or less frequent abnormal cardiovascular function test results.

**Conclusions:**

Male sexual function and autonomic control of the cardiovascular system are nearly normal in Fabry patients, which cast doubt on the general accepted assumption that autonomic neuropathy is the main cause of symptoms and signs compatible with autonomic dysfunction in Fabry disease. Possibly, end-organ damage plays a key role in the development of symptoms and signs in Fabry patients. An exceptional kind of autonomic neuropathy is another but less likely explanation.

## Background

Fabry disease (OMIM 301500) is an X-linked glycolipid storage disease caused by deficient activity of the lysosomal enzyme α-galactosidase-A leading to lysosomal accumulation of globotriaosylceramide and subsequently to severe multi-system disease. Male patients are usually severely affected, but symptoms and signs often appear also in female carriers. They consist of skin lesions (angiokeratomas), corneal opacities, cardiac hypertrophy and rhythm disturbances, renal failure, acroparesthesias, defective sweating (hypo- or anhidrosis), abdominal pain and diarrhoea.

Abnormalities of tears and saliva formation, cardiac rhythm disturbances, defective sweating and gastrointestinal complaints have frequently been explained by autonomic failure. As several studies have shown small fibre damage [1,2] and accumulation of lipids in the autonomic ganglia in Fabry patients [3], symptoms and signs compatible with autonomic dysfunction have generally been attributed to autonomic neuropathy [4-6]. However, more recently, an- or hypohydrosis has been found to be due to sweat gland dysfunction rather than autonomic neuropathy [7].

Autonomic involvement occurs in a wide range of peripheral neuropathies, albeit with variable symptomatology. Orthostatic intolerance and male sexual dysfunction, however, are invariably found in autonomic neuropathies [8]. Surprisingly, these symptoms are infrequently reported in Fabry disease. Symptom surveys that systematically investigated the presence of orthostasis and impotence in Fabry patients are lacking. Data obtained from the Fabry Outcome Survey (FOS), a European outcomes database of clinical manifestations in Fabry patients, revealed only a limited number of cases with orthostatic intolerance [9], while male sexual dysfunction was not recorded at all. Only few studies investigating cardiovascular autonomic function have been carried out in Fabry patients. Heart rate variability has been tested in small numbers of Fabry patients and showed abnormal results in two studies [10,11] while others did not find decreased heart rate variability [12]. None of the studied Fabry patients had orthostatic hypotension [6,12].

Altogether, symptoms and signs compatible with autonomic dysfunction in Fabry disease are considered to be due to autonomic neuropathy, while studies focusing on typical symptoms and signs are scarce. The main objective of this study was to investigate the presence of autonomic neuropathy with emphasis on autonomic symptomatology and impaired cardiovascular autonomic control in Fabry patients.

## Methods

The Academic Medical Center (AMC) is the single referral center for the treatment of Fabry patients in the Netherlands. All male and female Fabry patients aged 12 years and older who visit the outpatient pediatric or adult clinic for inherited metabolic diseases at the AMC were asked to participate. In all patients a diagnosis of Fabry disease was confirmed by enzymatic assay or DNA mutation. The study was approved by the local Ethics Committee and all patients (and parents if applicable) provided written informed consent.

To measure the severity of Fabry disease in individual patients, the Mainz Severity Score Index (MSSI) was used [13]. Patients with a total score of less than 20 are defined to be mildly affected, those with a score from 20-40 are moderately affected, and those with a score above 40 are severely affected [14]. Pain intensity was assessed on an 11-point visual analogue scale (VAS), anchored no pain (0) and worst possible pain (10). Patients were asked to score their most severe pain in the last 4 weeks [15].

Presence and severity of autonomic symptoms were assessed using the Autonomic Symptom Profile (ASP) [16]. This questionnaire consists of 73 items on different aspects of autonomic dysfunction and has 11 weighted subscale scores, including an orthostatic intolerance and a male sexual dysfunction subscale. The total score is the calculated sum of the 11 individual subscales, giving a maximum score of 200 for males and of 170 (200 minus 30 for the male sexual dysfunction scale) for females. A score of zero means no complaints. A higher score indicates more or worse symptoms. Originally this instrument was tested in three different groups: healthy controls, patients with all kinds of non-autonomic peripheral neuropathy and patients with proven autonomic neuropathy [16]. Healthy controls had a mean sum score of 10, patients with non-autonomic peripheral neuropathy had a mean score of 26, and patients with proven autonomic neuropathy had on average a score of 52. The latter scored on average 21.6 (score more than zero: 90%) in the orthostatic intolerance domain and 9.5 (score more than zero: 71%) in the male sexual dysfunction domain. Scores obtained from our patients were compared with these scores and with scores obtained from a sample of 48 sex- and age-matched healthy hospital workers who were asked for their co-operation by one of the authors (MB).

Furthermore, patients underwent cardiovascular autonomic function tests that were based on the guidelines designed for the detection of diabetic autonomic dysfunction and formulated by diabetes specialists in 1992 at the San Antonio Conference on Diabetic Neuropathy [17]. According to this conference an abnormality on more than one test is desirable to establish the presence of autonomic dysfunction.

In this study a forced breathing test and a standing up test from supine were performed. Changes in heart rate and blood pressure induced by forced breathing and standing up were assessed and compared to control values for these changes. Lower limits of normal (2.5 percentile) per age-group have been well-established in our laboratory [18]. The forced breathing test was performed in supine position. After 5 minutes rest the patient was instructed to perform six consecutive maximal inspiration and expiration cycles at a rate of 6 breaths per minute. To quantify the test score, the difference between maximal and minimal heart rate for each of the six cycles was determined and averaged to obtain the Inspiratory-Expiratory (I-E) difference in beats per minute [18]. After standing up, heart rate increases. The highest heart rate in the first 15 seconds from the onset of standing was determined and expressed as the increase from baseline (ΔHRmax). We used the highest and lowest heart rate in the first 30 seconds from the onset of standing to quantify the relative bradycardia (HRmax/HRmin ratio). Heart rate and finger blood pressure at 3 minutes after the change of posture were measured. A persistent fall of more than 20 mmHg in systolic pressure after 3 minutes standing and a fall of more than 10 mmHg in diastolic pressure after 3 minutes standing were considered to be abnormal.

### Statistical analysis

All results are expressed by mean and standard deviation or median and range where appropriate. Differences between variables are calculated using the unpaired t-test or Mann Whitney test and differences in proportions are tested using the Fisher's exact test. Correlations between variables are described with the use of Spearman correlation coefficients (Spearman's rho). Significance is defined at a p-value of < 0.05.

## Results

A total of 70 Fabry patients are followed at regular intervals at the outpatient pediatric or adult clinic for inherited metabolic diseases at the AMC. All patients were asked to participate in the study. Two adult patients could not be reached by phone and 15 adult patients refused to participate. Travel distance was the most frequently mentioned reason. Another 5 children did not take part in the study, mostly as their parents considered participation as too demanding. Half of the 22 non-participating patients were male. The median age was significantly lower than that of the participating patients (29 years, p = 0.008) but the MSSI was not different (median sum score 12.5, p = 0.74) at the time the study started.

In total, 48 patients (69% of the Dutch Fabry population) were included. In Table 1 the patient characteristics are shown. Thirty patients received enzyme replacement therapy (ERT) with a median duration of 3.6 years (range 0.2-6.0). Other frequently prescribed medications were: pain medication (11 patients of whom 1 used non-steroidal pain medication), ACE inhibitors (10 patients), ATII-antagonists (11 patients), beta blockers (3 patients) and diuretics (9 patients).

**Table 1:** Patient characteristics.

	All patients n = 48	Male patients n = 15	non-ERT n = 2	ERT n = 13	non-ERT versus ERT, p-value	Female patients n = 33	non-ERT n = 16	ERT n = 17	non-ERT versus ERT, p-value
Age, years median (range)	47 (12-73)	47 (21-66)	47.5 (46-49)	47 (21-66)	1.00	45 (12-73)	25.5 (12-52)	51 (22-73)	**0.00**

Enzyme activity, μmol/l/h median (range)	14 (0.0-59)	1.2 (0.0-6.4)	2.0 (1.5-2.5)	0.7 (0.0-6.4)	0.20	19.6 (4.1-59.3)	20 (13-51)	19 (4.1-59)	0.51

MSSI sum score median (range)	15.5 (1-47)	25 (5-47)	11 (5-17)	27 (10-47)	0.23	13 (1-35)	7 (1-18)	23 (3-35)	**0.00**
general score median (range)	4 (0-11)	5 (2-11)	3.5 (2-5)	5 (2-11)	0.31	4 (0-8)	2 (0-5)	5 (1-8)	**0.00**
neurological score median (range)	4 (0-13)	6 (0-13)	0.5 (0-1)	6 (3-13)	**0.02**	3 (0-12)	1.5 (0-7)	4 (1-12)	**0.04**
cardiovascular score median (range)	6.5 (0-15)	9 (1-15)	7 (3-11)	9 (1-15)	0.69	4 (0-15)	1.5 (0-11)	9 (0-15)	0.07
renal score median (range)	0 (0-12)	0 (0-12)	0	0 (0-12)	0.38	0 (0-12)	0	4 (0-12)	**0.00**

VAS most severe pain median (range)	3 (0-9)	4 (0-8)	1	4 (0-8)	0.31	2 (0-9)	0.5 (0-8)	5 (0-9)	0.20

	**n = 36**	**n = 10**	**n = 2**	**n = 8**		**n = 26**	**n = 9**	**n = 17**	

Systolic BP, mmHg mean (SD)	118 (12)	114 (13)	115 (20)	113 (13)	0.88	120 (11)	115 (8)	123 (12)	0.11

Diastolic BP, mmHg mean (SD)	71 (7)	71 (9)	74 (16)	70 (8)	0.68	71 (7)	70 (9)	72 (5)	0.46

Heart rate, bpm mean (SD)	65 (12)	68 (13)	61 (4)	70 (14)	0.40	64 (12)	66 (13)	63 (11)	0.47

ERT = enzyme replacement therapy, MSSI = Mainz Severity Score Index, VAS = visual analogue scale, BP = blood pressure, bpm = beats per minute

The MSSI sum score revealed that on average male patients were moderately affected and females were mildly affected. Twenty-nine patients scored below 20 (mildly affected), 18 patients scored between 20-40 (moderately affected) and only 1 male patient had a sum score of more than 40 (severely affected) [14]. The median score for most severe pain in the last 4 weeks was 3 (range 0-9). This score did not differ significantly between male and female patients.

All patients completed the ASP. Table 2 shows median scores per domain. In Table 3 the numbers of patients with a score of more than zero for the different domains are shown. The median ASP sum score for all patients was 22 (range 0-78). Male patients had a median score of 18 out of 200 points and female patients scored 24 out of 170 points. Neither these scores (p = 0.71), nor the scores of the different domains differed between males and females. Controls (15 males, 33 females, median age 46 years, range 14-74) had a median score of 12 (range 0-35), which was better than Fabry patients (p = 0.005).

**Table 2:** Autonomic Symptom Profile scores, median (range).

Domain	All patients n = 48	non-ERT n = 18	ERT n = 30	non-ERT versus ERT, p-value	Healthy controls n = 48	Patients versus controls, p-value
Orthostatic intolerance	13 (0-35)	14 (0-28)	13 (0-35)	0.96	1.3 (0-25)	**0.004**

Vasomotor impairment	0 (0-8.2)	0 (0-7.6)	0 (0-8.2)	0.33	0 (0-6.3)	**0.017**

Secretomotor disorder	1.5 (0-14)	0 (0-7.5)	3 (0-14)	**0.000**	1.5 (0-7.5)	0.23

Gastroparesis	0 (0-8.4)	0 (0-8.4)	0 (0-5)	0.36	0 (0-3.3)	**0.017**

Diarrhoea	0 (0-16)	0 (0-8)	0 (0-16)	0.57	0 (0-8)	1.00

Constipation	0 (0-11)	0 (0-6)	0 (0-11)	0.95	0 (0-9)	0.63

Bladder disorder	0 (0-12)	0 (0-10)	0 (0-12)	0.38	0 (0-4)	0.74

Pupillomotor impairment	0.5 (0-4.5)	0.5 (0-2.5)	0.5 (0-4.5)	0.78	0 (0-3)	0.13

Sleep disorder	1.5 (0-7.5)	0 (0-6)	1.5 (0-7.5)	**0.002**	0 (0-6)	0.06

Syncope	0 (0-4)	0 (0-4)	0 (0-4)	0.29	0 (0-4)	0.31

Sum score	22 (0-78)	21 (0-49)	24 (0-78)	0.30	12 (0-35)	**0.005**

	**Male patients** **n = 15**	**non-ERT** **n = 2**	**ERT** **n = 13**	**p-value**	**Male controls** **n = 15**	**p-value**

Male sexual failure	0 (0-17)	0.8 (0-1.5)	0 (0-17)	0.93	0 (0-7.5)	0.29

ERT = enzyme replacement therapy

**Table 3:** Autonomic Symptom Profile Scores, score > 0, n (%).

Domain	All patients n = 48	non-ERT n = 18	ERT n = 30	non-ERT versus ERT, p-value	Healthy controls n = 48	Patients versus controls, p-value
Orthostatic intolerance	36 (75)	13 (72)	23 (77)	0.74	24 (50)	**0.02**

Vasomotor impairment	15 (31)	4 (22)	11 (37)	0.35	6 (13)	**0.047**

Secretomotor disorder	28 (58)	3 (17)	25 (83)	**0.00**	25 (52)	0.68

Gastroparesis	15 (31)	4 (22)	11 (37)	0.35	6 (13)	**0.047**

Diarrhoea	13 (27)	6 (33)	7 (23)	0.51	15 (31)	0.82

Constipation	10 (21)	4 (22)	6 (20)	1.00	14 (29)	0.48

Bladder disorder	17 (35)	5 (28)	12 (40)	0.54	18 (38)	1.00

Pupillomotor impairment	29 (60)	12 (67)	17 (57)	0.55	19 (40)	0.07

Sleep disorder	28 (58)	5 (28)	23 (77)	**0.00**	23 (48)	0.41

Syncope	3 (6)	2 (11)	1 (3)	0.55	1 (2)	0.62

Sum score	46 (96)	17 (94)	29 (97)	1.00	46 (96)	1.00

	**Male patients** **n = 15**	**non-ERT** **n = 2**	**ERT** **n = 13**	**p-value**	**Male controls** **n = 15**	**p-value**

Male sexual failure	6 (40)	1 (50)	5 (38)	1.00	3 (20)	0.43

ERT = enzyme replacement therapy

Median scores in the orthostatic intolerance domain were 13 (range 0-35) for Fabry patients and 1.3 (range 0-25) for healthy controls (p = 0.004). Seventy-five percent of the Fabry patients scored more than zero points in this domain compared with 50% of the healthy controls (p = 0.02). Median scores in the male sexual failure domain were 0 (range 0-17) for Fabry patients and 0 (range 0-7.5) for healthy controls (p = 0.29). Forty percent of male Fabry patients scored more than zero points in this domain compared with 20% of healthy male controls (p = 0.43). Furthermore, unexpectedly low numbers of Fabry patients scored more than zero points in the secretomotor, gastroparesis, diarrhoea and constipation domains, being respectively 28, 15, 13 and 10.

Spearman correlation did not show a relationship between age and the ASP sum score (Spearman's rho = 0.14, p = 0.35) and a trend towards an association between the MSSI sum score and ASP sum score was found (Spearman's rho = 0.27, p = 0.06).

**Figure 1:**
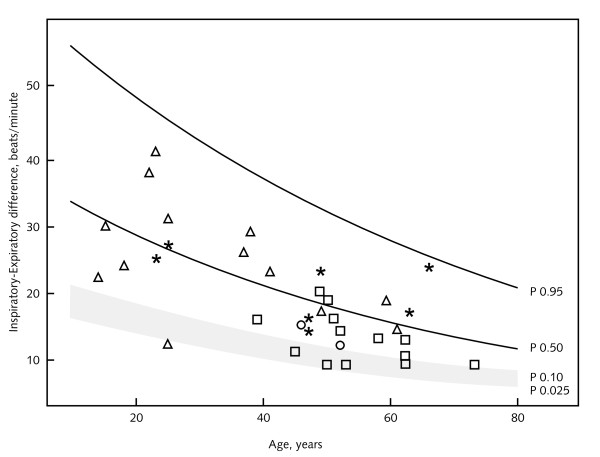
**Inspiratory-Expiratory difference**. Circle = male without LVH/cardiomyopathy. Star = male with LVH/cardiomyopathy. Triangle = female without LVH/cardiomyopathy. Square = female with LVH/cardiomyopathy.

Thirty-six patients (10 males and 26 females, 75% of the study population) underwent autonomic cardiovascular function tests. Twelve patients refused to make an extra visit to the hospital for the purpose of this study. In two cases heart rate variability (HRV) was impossible to assess due to frequent extrasystoles. The results of these tests are summarized in Table 4 and Figure 1.

**Table 4:** Autonomic function tests.

	n	Mean (SD)	Number of patients with an abnormal test result (n)
Inspiratory-Expiratory (I-E) difference bpm	34	19 (8)	3

ΔHRmax bpm	34	25 (10)	3

HRmax/HRmin ratio mmHg	34	1.3 (0.21)	0

ΔSyst 3 min after standing up mmHg	36	3 (9)	0

ΔDiast 3 min after standing up mmHg	36	6 (7)	1

ΔHR 3 min after standing up bpm	36	7 (7)	0

bpm = beats per minute

Three female patients aged 25, 50 and 53 years old and with MSSI sum scores of 13, 24 and 17 showed abnormal results of the forced breathing test. They scored respectively 12, 9 and 9 beats per minute indicating a decreased HRV. The ECG of the 50-year-old woman showed signs of left ventricular hypertrophy (LVH) and the 53-year-old woman suffered from cardiomyopathy. Three males aged 25, 52 and 63 years old and with MSSI sum scores of 13, 27 and 47 had an abnormal initial heart rate response to standing up from supine position. The first was known with LVH and the latter with cardiomyopathy, while the 52-year-old male did not have cardiac pathology. They scored respectively 17, 11 and 11 beats per minute which are abnormally low scores in relation to their age. One 14-year-old girl with an MSSI sum score of 2 had a persistent fall of 11 mmHg in diastolic pressure 3 minutes after standing up.

### Role of ERT

Two of the 15 male study patients were untreated. They were similar to ERT treated patients with respect to age and disease severity. Female patients who were treated with ERT were older and more severely affected compared with untreated females (see Table 1). No difference in ASP sum score was found between ERT treated and untreated patients (p = 0.30), while secretomotor problems and sleep disorder were more frequent and more severe in ERT treated than untreated patients (see Tables 2 and 3).

Twenty-five of the 36 patients (69%) who underwent autonomic cardiovascular function tests received ERT. Patients on ERT had lower heart rate variability than untreated patients. All three females and three males with an abnormal heart rate variability test result were treated with ERT. The 14-year-old girl was untreated. Altogether, ERT was not associated with less severe disease, lower ASP scores or less frequent cardiovascular autonomic function abnormalities.

## Discussion

In this study we found a low prevalence of orthostatic intolerance and male sexual dysfunction in a rather large cohort of Fabry patients. The cardiovascular autonomic function tests showed normal cardiovascular autonomic control in almost all of our Fabry patients.

This raises the important question whether autonomic neuropathy plays a prominent role in Fabry symptoms and signs. The disturbances that have been ascribed to autonomic neuropathy in Fabry disease include an- or hypohydrosis, decreased tears and saliva formation, abnormal cerebrovascular reactivity, cardiac rhythm disturbances as well as gastrointestinal complaints [4-6]. However, unlike the findings in other diseases that cause autonomic neuropathy [8] and in patients with proven autonomic failure [16], orthostatic intolerance and male sexual dysfunction are less frequent and less severe manifestations in our Fabry patients. Furthermore, the low resting heart rate as found in our study patients is unusual in patients with an autonomic neuropathy.

Defective sweating has long been thought to originate from autonomic neuropathy. However, skin biopsies did not reveal a decrease in nerve fibre density of sweat gland innervation, but revealed storage of lipids in sweat glands. Also, the non-length dependent distribution of the an- or hypohydrosis and the rapid effect of single enzyme infusions, suggested a sweat gland dysfunction rather than an autonomic neuropathy [7]. In line with defective sweating, end-organ failure might also account for the abnormal peripheral blood flow that has been found in several studies [19,20]. A study on vascular hyperreactivity in Fabry disease supports this hypothesis; absence of a difference in plasma epinephrine or norepinephrine levels between patients and controls suggested that the altered vessel response in Fabry disease may be attributed to vasogenic and not to neurogenic factors [21]. This is further supported by almost normal cardiovascular autonomic function, as has been confirmed in the current study as well as in previous studies [6,12]. Studies on heart rate variability (HRV) in pediatric Fabry patients revealed significantly different results between boys and both girls and controls, with significant improvement of heart rate variability in boys upon ERT [10,11]. However, it is likely that cardiac pathology (i.e. left ventricular hypertrophy and/or conduction system pathology) has influenced the abnormalities observed in these patients. In the current study, only 6 out of 36 Fabry patients showed an abnormal HRV on one test. As four of these six patients were known with LVH or cardiomyopathy, our findings could be partly explained by the underlying cardiac pathology.

Altogether, our results indicate that symptoms and signs compatible with autonomic dysfunction in Fabry patients are probably not due to autonomic neuropathy. More likely, these symptoms and signs are caused by end-organ failure which has been suggested before by others [22,23] and is supported by findings from previous studies [7,21]. An exceptional kind of autonomic neuropathy can, however, not be totally excluded.

It is surprising that, in spite of evidence of small fibre neuropathy in Fabry disease [1,2], and autonomic functions being carried by these small nerve fibres, we did not find symptoms and signs that are generally found in patients with autonomic neuropathy due to other diseases. Diabetic neuropathy and other small fibre neuropathies (i.e. amyloidosis, leprosy and HIV) lead to equal damage of C and Aδ fibres or to more severe damage of C fibres than Aδ fibres [24-27]. In contrast, Fabry disease causes more severe impairment of Aδ fibres compared to C fibres, as shown by quantitative sensory testing [1,12]. Preganglionic autonomic fibres consist of small myelinated B fibres and postganglionic autonomic fibres are small unmyelinated C fibres [28]. The relatively selective damage to 'non-autonomic' small myelinated Aδ fibres in Fabry disease could therefore underlie the found preservation of autonomic function in Fabry disease. One other disease, hereditary sensory and autonomic neuropathy (HSAN) type 5, is known to cause selective loss of small myelinated nerve fibres. Autonomic function is usually spared in HSAN type 5; none of the reported patients had orthostatic hypotension, although anhydrosis has been reported in some [29]. Apparently, damage to C-fibres is required for the development of overt autonomic neuropathy.

In contrast to orthostatic intolerance and male sexual dysfunction, gastrointestinal complaints are frequently reported in Fabry disease. These complaints have been attributed to dysfunction of enteric neurons [30]. Data obtained from 342 Fabry patients enrolled in the Fabry Outcome Survey (FOS), revealed that 60.8% of children and 49.8% of adults experienced gastrointestinal complaints. The most frequently reported gastrointestinal symptoms were abdominal pain and diarrhoea [30]. In this light, the high numbers of patients without gastrointestinal disturbances in the current cohort are unexpected. This difference between the FOS data and our results may be due to an overestimation in the FOS as these findings were based on self-reports by patients, and not on a validated questionnaire.

Furthermore, an unexpectedly low number of Fabry patients reported secretomotor problems. This may be attributed to a shortcoming in the questionnaire we used: the ASP assesses changes in sweating in the past five years, whereas Fabry patients usually suffer from sweat problems from childhood on.

Another limitation of the current study is that we included mainly mild to moderately affected patients. As we found a trend towards a correlation between the MSSI sum score and the ASP sum score and relatively high MSSI sum scores in 4 out of 6 patients with an abnormal heart rate variability test result, we cannot exclude that autonomic neuropathy is more prevalent in severely affected patients. Besides, the cross-sectional character of the study precludes definite conclusions on the long term effect of enzyme replacement therapy in individual patients. However, our results suggest that patients with relatively severe disease are more often on ERT and that the severity of autonomic dysfunction is not influenced by ERT. A final limitation is that we have restricted the function tests to those evaluating cardiovascular autonomic function. However, abnormalities in the autonomic control of other organ systems such as peripheral vascular reactivity is thought to reflect end-organ pathology and not real autonomic neuropathy as discussed above.

## Conclusions

Male sexual function and autonomic control of the cardiovascular system are normal in Fabry patients, which cast doubt on the general accepted assumption that autonomic neuropathy is a major player in the pathophysiology of the disease.

## Competing interests

MB received research support from Actelion Pharmaceuticals Ltd.

INvS received honoraria for lecturing and consultancy and research support from Actelion Pharmaceuticals Ltd. All consulting fees for INvS were donated to the Stichting Klinische Neurologie, a local foundation that supports research in the field of neurological disorders.

WW declares that he has no competing interests.

FAW received reimbursement of expenses and honoraria for lectures on lysosomal storage diseases, including Fabry disease, from Genzyme Corporation, Actelion Pharmaceuticals Ltd and Shire HGT.

CEMH received reimbursement of expenses and small honoraria for lectures on the management of lysosomal storage diseases, including Fabry disease, from Genzyme Corporation, Actelion Pharmaceuticals Ltd and Shire. All honoraria were donated to the Gaucher Stichting, a national foundation that supports research in the field of lysosomal storage disorders.

## Authors' contributions

MB participated in the design and coordination of the study, performed the statistical analyses and drafted the manuscript. INvS, FAW and CEMH conceived of the study and participated in its design and in drafting the manuscript. WW carried out the cardiovascular autonomic function tests and contributed to the interpretation of the results. All authors read and approved the final manuscript.

## Pre-publication history

The pre-publication history for this paper can be accessed here:

http://www.biomedcentral.com/1471-2377/10/38/prepub
